# Communicative Participation in Dysarthria: Perspectives for Management

**DOI:** 10.3390/brainsci12040420

**Published:** 2022-03-22

**Authors:** Allyson D. Page, Kathryn M. Yorkston

**Affiliations:** 1 School of Communication Sciences and Disorders, Western University, London, ON N6G 1H1, Canada; 2 Department of Rehabilitative Medicine, University of Washington, Seattle, WA 98105-6246, USA; yorkston@uw.edu

**Keywords:** dysarthria, communicative participation, patient reported outcome measures

## Abstract

Communicative participation is restricted in many conditions associated with dysarthria. This position paper defines and describes the construct of communicative participation. In it, the emergence of this construct is reviewed, along with the predictors of and variables associated with communicative participation in the dysarthrias. In doing so, the features that make communicative participation unique and distinct from other measures of dysarthria are highlighted, through emphasizing how communicative participation cannot be predicted solely from other components of the World Health Organization’s International Classification of Functioning, Disability and Health (ICF), including levels of impairment or activity limitations. Next, the empirical literature related to the measurement of communicative participation and how this research relates to dysarthria management is presented. Finally, the development of robust clinical measures of communicative participation and approaches to management is described from the point of view of the clinician. We argue that communicative participation should be a primary focus of treatment planning and intervention to provide patient-centered, holistic, and value-based clinical interventions which are responsive to the needs of individuals living with dysarthria.

## 1. Introduction

Clients who have undergone speech treatment can provide a rich source of information, as they provide their unique perspectives on management of dysarthria. One man with Parkinson’s disease (PD) suggested, “The part I liked best is the conversation part … that’s actually using speech and being heard in a meaningful sense. The trouble with *aah* (the exercises) is it doesn’t mean anything” [1] (p. 213). This quote reminds us that improving the sound of speech is not the only goal of intervention. Speaking must be viewed within the social context of communication. This position paper defines and describes the construct of communicative participation. In it, the emergence of this construct will be reviewed, along with the features that make it unique and distinct from other measures of dysarthria. This article will also review the literature concerning research related to the measurement of communicative participation and how it is associated with other aspects of dysarthria. Finally, approaches to management will be described from the point of view of the clinician.

### Definition and Description

Eadie and colleagues defined communicative participation as “taking part in life situations where knowledge, information, ideas, or feelings are exchanged” [2] (p. 309). This construct can best be understood by reviewing how the field of dysarthria management has developed. The modern era of dysarthria management began in the 1960s with the seminal work of Darley, Aronson, and Brown [3,4]. They listened to samples of speech representing various medical conditions associated with dysarthria. In doing so, they classified and identified six types of dysarthria (flaccid, spastic, ataxic, hypokinetic, hyperkinetic, and mixed), based on clusters of salient auditory-perceptual features associated with lesions in the central and peripheral nervous systems unique to each dysarthria type [3,4], and demonstrated that the dysarthrias represent a diverse group of motor speech disorders. The methods developed by Darley, Aronson, and Brown continue to be the basis of different diagnoses of the dysarthrias.

The next major phase of development contributing to our understanding and management of dysarthria came from those who sought to understand the physiology of speech production [5,6,7,8,9,10]. These researchers suggested that dysarthria was not merely an ‘articulation’ disorder, but should be examined through an in-depth understanding of respiratory, phonatory, laryngeal, and velopharyngeal components of speech production, in addition to aspects of oral production. This physiologic method led to many advancements in treatment approaches that sought to reduce the impairment associated with physiologic components of dysarthric speech production. The outcomes of speech treatment using a physiological approach could be measured not only by the improvement of speech components, but also by overall measures of speech adequacy, such as measures of speech intelligibility.

The next pivotal era of dysarthria management was marked by the development of the World Health Organization’s, International Classification of Functioning, Disability and Health (ICF) [11]. The ICF is a conceptual framework of disability based on a bio-psychosocial model of health. The ICF defines the construct of ‘impairment’ as a “problem in body function or body structure”. This corresponds to the physiological components of speech production. The ICF also includes the construct of ‘activity’ or the “execution of a task or action by an individual”. Activity can be equated to the task of speaking. Finally, and perhaps the most ground-breaking, is the construct of ‘participation’ or “involvement in life situations” [11]. In short, the management of dysarthria has moved and evolved from the medical model of the 1960s to a more bio-psychosocial perspective.

The introduction of the ICF in 2001 was instrumental in allowing for the study of communicative participation as a distinct construct situated within a theoretical framework. The construct of communicative participation is unique from other aspects of dysarthria for several reasons. The first reason is that it must be viewed within a social context. To illustrate this, consider how the construct of communicative participation is different from the other constructs of the ICF model. The ‘activity’ of speaking is often assessed from audio-recorded speech samples based on standard passages. The focus of this approach is on the perceptual or acoustic properties of the speech sample, and speech production can be assessed without any information about the social context. With the focus on the ‘activity’ of speaking, the intent of communication, the environment, and communication partners are relatively unimportant. Similar to the management of dysarthria from a physiologic impairment perspective, this approach focuses on the assessment of speech subsystem impairment. Treatment is conducted in a clinic room, and requires no social context. The next distinct characteristic of the construct, communicative participation, is that it must be evaluated from the perspective of the person with dysarthria. For example, while the clinician is the expert in assessing, for example, velopharyngeal function, only the speaker with dysarthria has a full picture of his or her social context. Finally, the construct of communicative participation is unique from impairment and activity-based constructs because participation may be similar across diverse neurologic conditions. For example, although the speech impairment associated with Parkinson’s disease is distinctly different from the speech impairment associated with brainstem stroke, there may be similar restrictions in communicative participation experienced across these clinical populations. Future research is needed to understand whether issues related to communicative participation (e.g., isolation, embarrassment, burden on close partners) share more similarities than differences across the dysarthrias.

## 2. Exploration of Communicative Participation in the Dysarthrias

In the previous section, we suggested that the ICF conceptual framework expanded the ability to research a critically understudied aspect of dysarthria—how communicative participation is experienced by those living with dysarthria. In the sections that follow, the current state of knowledge relating to communicative participation in the adult dysarthrias will be described, and, in doing so, the barriers to and predictors of communicative participation in dysarthria will be highlighted. Next, patient-reported outcome measures that assess communicative participation will be described, the importance of the continued study of communicative participation in dysarthria research will be emphasized, and concrete guidance will be provided for clinicians interested in conducting participation-based interventions. Finally, it will be argued that speech-language pathologists have a unique role to advocate for and champion meaningful, comprehensive, and holistic care through the inclusion of communicative participation outcomes in the management of individuals with dysarthria.

### 2.1. A Distinct Construct

There has often been an assumption made by both clinicians and researchers that treatment effects obtained within the clinical setting directly translate to successful communicative participation [12]. There has also been an assumption that the severity of dysarthria is predictive of the severity of the restrictions to communicative participation [13]. Several authors have underscored the importance of exploring the relationship between dysarthria severity and perceived communicative difficulties [14,15,16], and have cautioned that, in the absence of assessment data on communicative participation, there has been a tendency to make clinical inferences about the effectiveness of dysarthric communication based on objective clinical measures such as instrumental, physiologic, acoustic, or auditory-perceptual measures [13,17,18,19]. A growing body of empirical research provides confirmation that communicative participation is likely a distinct construct, and that this construct cannot necessarily be predicted from the severity of the communication disorder. A component of communicative participation, communicative effectiveness, is included in this empirical literature, and is defined as a person’s ability to successfully communicate messages in home and community settings to fulfil life roles [15].

Several studies have sought to correlate impairment-based (i.e., physiologic, acoustic) or activity-based (i.e., speech intelligibility) outcome measures with participation-based outcomes [12,17,18,19]. Dykstra (Page) and colleagues studied 30 individuals with Parkinson’s disease and hypophonia, and found a weak, non-significant correlation between speech intensity and self-rated communicative effectiveness [17], as measured by the Communicative Effectiveness Index (CETI) [20]. Additionally, Ball and colleagues studied 25 individuals with amyotrophic lateral sclerosis (ALS) using the CETI, and found that participants rated dimished communicative effectiveness with only minor reductions in speech intelligibility [18]. McAuliffe and colleagues, and Donovan et al. did not find significant relationships between speech intelligibility and self-rated communicative effectiveness in participants with traumatic brain injury (TBI) and PD, respectively [12,19]. There are studies however, proposing that impairment and activity-based outcomes are correlated with communicative participation [21,22]. Borrie and collegues studied 32 participants with various etiologies of dysarthria, and observed a moderate relationship between articulatory precision and communicative participation, mediated primarily through speech intelligibility [21]. Similarly, Sixt Börjesson et al. administered the CPIB to 30 individuals with ALS, and reported a strong postive correlation between speech intelligibility and CPIB scores [22]. Taken together, these results suggest that communicative participation is complex, likely a distinct construct, and in need of further investigation.

### 2.2. Barriers to Communicative Participation

With the study of communicative participation being a relatively new and emerging field in speech-language pathology, several research groups have sought to understand this construct by identifying barriers that contribute to restricted communicative participation from both quantitative and qualitative perspectives [17,23,24].

Several studies have sought to identify the communicative situations and contexts that can serve as barriers to successful and effective communication for individuals with different dysarthria types [17,23,24]. Dykstra (Page) and colleagues administered the Communicative Effectiveness Survey (CES), an eight-item patient-reported outcome measure, to 10 speakers with oromandibular dystonia (OMD) and hyperkinetic dysarthria [23]. Despite participants presenting with milder speech intelligibility deficits (average sentence intelligibility: 90.91%, Speech Intelligibility Test [25]), self-perceived reductions in communicative effectiveness were reported as barriers across a range of communicative contexts and situations, including conversing with a stranger or a familiar person on the telephone, having a conversation with a family member or friend at home, having a conversation while traveling in a car, and participating in a conversation with strangers in a quiet place [23]. Dykstra et al. also explored self-rated communicative effectiveness by administering the CES to 30 individuals with PD and hypophonia [17]. Conversing over a distance and having a conversation while traveling in a car were reported as barriers to effective communication [17]. Garcia et al. explored the perceived barriers to work reintegration of people with a variety of communication disorders, including 13 individuals with dysarthria [24]. Individuals with dysarthria reported barriers related to communicating a message forcefully, having to speak efficiently and accurately, and being able to get a point across convincingly while in a work setting. Difficulty communicating in noisy settings, speaking on the telephone, interacting in large meetings, and speaking to strangers and to people in positions of authority were also reported as barriers to successful communication [24].

### 2.3. Predictors of Communicative Participation

The study of communicative participation has also been approached from the perspective of identifying predictors of communicative participation within and across different dysarthria types. Using the Communicative Participation Item Bank—a 10-item short form (CPIB) [26], McAuliffe and colleagues explored predictors of communicative participation in 378 individuals with PD living in New Zealand and the United States [27]. Overall, the strongest predictor of restricted communicative participation was greater perceived speech impairment. Lower levels of speech usage, cognitive symptoms, emotional issues, fatigue, and swallowing difficulties were also identified as predictors to reduced communicative participation [27]. In another study, also using the CPIB, Yorkston and colleagues explored variables associated with communicative participation in 70 individuals with ALS who still used natural speech to communicate [28]. The variables found to be most strongly correlated with restricted communicative participation were self-reported speech severity, swallowing severity, and lower levels of speech usage [28]. Yorkston and others administered the CPIB to 216 individuals with multiple sclerosis (MS), reporting communication problems to examine the extent to which demographic and symptom-related different variables contributed to restricted communicative participation [29]. Results indicated that reduced cognitive and speech skills, self-reported increased speech severity, lower levels of speech usage, limitations in physical activity, and higher levels of education were significant predictors of restricted communicative participation [29]. Similarly, Baylor and colleagues explored variables associated with self-reported communicative participation in 498 individuals with MS [30]. A total of six variables were identified that were significantly associated with communicative participation. These variables, listed in order from strongest to weakest associations were, fatigue, slurred speech, depression, problems thinking, employment status, and perceived social support. Increased problems thinking, slurred speech, depression, and fatigue were associated with greater restrictions to communicative participation, while increased social support and involvement in paid employment were associated with fewer restrictions to communicative participation [30].

### 2.4. Similarities across Populations

Restrictions in communicative participation occur commonly in all dysarthria types. Several studies have explored communicative participation in participant groups sharing a single aetiology (i.e., PD, MS, ALS, OMD), but there have been efforts to explore if commonalities exist in communicative participation across dysarthria types. Jin and colleagues explored if there were common variables that predicted communicative participation across four communication disorder diagnoses that represented mostly motor speech and voice disorders [31]. Jin administered the CPIB, along with several psychosocial-based patient-reported outcome measures, to participants with PD and hypokinetic dysarthria, dysarthria due to cerebrovascular accident (CVA), spasmodic dysphonia (SD), and vocal fold immobility (VFI) to determine commonalities or differences in variables predictive of communicative participation across the diagnostic groups. Self-rated speech/voice severity and self-rated effort were found to be the most predictive factors of communicative participation across diagnostic groups. Mental health, perceived social support, and resilience also contributed to variance in communicative participation when pooled across diagnoses. There were differences between some diagnostic groups, however, with the VFI group having the most restricted CPIB scores in comparison to the other diagnostic groups [31].

### 2.5. Perspectives of Speakers with Dysarthria

Qualitative research methods have also significantly contributed to our understanding of how communicative participation is experienced by individuals with dysarthria. One such methodology, called phenomenology, seeks to understand the insider’s experience of living with a communication disorder. The study of dysarthria from a phenomenological approach provides space for the ‘participant voice’, and can capture the complexities of a speech disorder by allowing the individual with dysarthria to be the ‘expert’, rather than the researcher or clinician [32]. Several qualitative studies have explored communicative participation in single dysarthria groups with shared aetiologies [13,33,34,35], while other studies have sought to understand if there are commonalities in how communicative participation is experienced across different communicative disorders [36,37]. Both approaches are important because they provide an understanding of disorder specific restrictions to communicative participation, and reveal potential commonalities across a variety of dysarthria types and aetiologies.

In a study that explored how changes in communication impact the lives of individuals with PD, Miller and colleagues interviewed 37 participants with PD who identified changes in their speech production [33]. Interestingly, the nature of participants’ speech and voice changes were not a main concern, but instead, the main concerns identified by participants related to how their communication skills affected their self-concept, family dynamics, and participation both inside and outside of their family. As a result, participants reported that they used a variety of coping strategies that facilitated others’ understanding, such as of physical strategies, monitoring and adjusting strategies, and managing conversations. The authors concluded that speech and voice changes impact the individual and the family well in advance of any noticeable reductions in speech intelligibility [33]. Yorkston and colleagues explored the experiences of individuals with MS and satisfaction with communicative participation using phenomenological methods and semi-structured interviews across two studies [13,34]. In the first study, participants reported that mild communicative impairments contributed to reduced communicative participation. However, these same participants also reported that non-speech factors, including fatigue, mobility limitations, visual impairment, and the unpredictability of communication, all contributed to restricted communicative participation [13]. In the second study, Yorkston and colleagues asked eight participants with MS to discuss their satisfaction with communicative participation in a variety of situations [34]. The results of this study revealed that satisfaction with communicative participation is multidimensional and involved comfort, success of outcome, and the personal meaning of participation [34]. In another study, also using a phenomenological approach, Page and colleagues interviewed eight individuals with OMD and dysarthria to explore the consequences of OMD on communicative participation from the ‘insider’s perspective’ [35]. Common themes to emerge from the face-to-face interviews related first to changes in speech production, such as slowed rate of speech, increased physical effort, and difficulty articulating certain speech sounds. Participants explained how changes in speech production affected their communicative participation in everyday life. Participants reported interferences to communicative participation due to the unpredictability of dystonic contractions and the corresponding impact on speech intelligibility, especially in unfamiliar and stressful situations. The second theme related to how OMD negatively impacted their roles in the workplace, at home, and in social activities. The third and final theme related to strategies participants used to carry on with their lives following their diagnosis. Strategies included family support, educating others, humour, and participating in alternate activities [35]. This study revealed the pervasive consequences of OMD that extend beyond the communication disorder [35].

There is an emerging body of qualitative research suggesting commonalities and similarities of how communicative participation is experienced by people with a variety of communication disorders [36,37]. In a qualitative study exploring the experience of living with dysarthria, Walshe and Miller interviewed 10 people with dysarthria of different aetiologies (cerebellar atrophy, motor neurone disease, stroke-related dysarthria, multi-system atrophy, PD, MS, Friedreich’s ataxia) [36]. This study demonstrated commonly shared psychological and social impacts for the speaker with dysarthria. Participants reported that they limited their amount of speaking, limited participation in unnecessary conversations, attempted to hide or conceal their communication disorder, and avoided certain words and communicative situations. Changes in communication style, relationships, leisure activities, lifestyle, social life, and increased social isolation were reported by participants with different dysarthria aetiologies demonstrating that that impact of dysarthria extends beyond the severity of the motor speech impairment [36]. Similarly, Baylor et al., explored the factors contributing to interference with communicative participation in adults with dysarthria of different aetiologies (SD, MS, PD, ALS) [37]. Despite differences in impairments and activity limitations, participants described similar communicative participation restrictions. The results of this study identified shared sources of interferences to communicative participation, in which participants defined interference as limitations in accomplishing tasks as well as the emotional consequences. Further, as a group, participants reported experiencing limited control over their symptoms and their environment, but they reported a greater sense of control over their personal decisions and priorities, which influenced their ability to participate [37]. Baylor and colleagues urged that we need to move beyond an impairment-driven perspective which assumes that the impairment underlying the communication disorder determines the interferences or restrictions to communicative participation, and instead explore how clinical interventions can facilitate communicative participation, regardless of the type of dysarthria or, more broadly, type of communication disorder [37]. Collectively, these studies highlight that restrictions to communicative participation can arise from many types of impairments, and not solely from impairments in speech production. Yorkston cautioned that if non-speech factors are not acknowledged, then our clinical interventions will be compromised [29].

Impact on communicative participation has also been studied following clinical interventions, such as botulinum toxin (BoNT) therapy for laryngeal dystonia (LD) and oromandibular dystonia [38,39]. Recently, Yorkston and colleagues explored the impact of BoNT injections on communicative participation for individuals living with LD [38]. Using phenomenological methods, Yorkston reported that, although many participants received benefits from BoNT, many participants also experienced persistent restrictions in communicative participation [38]. Page et al. explored the psychosocial impact of BoNT injections for individuals with OMD using phenomenological methods, and found that BoNT therapy had a variable impact on speech production, satisfaction with treatment, and communicative participation [39]. Both studies emphasize the importance of understanding the patient perspective regarding satisfaction and impact on communicative participation following a clinical intervention. Yorkston asserted that clinicians should not assume that some improvement in voice resulting from BoNT treatment is sufficient to remove participation restrictions. Instead, clinicians should enhance the outcomes of BoNT therapy with a participation-based approach to intervention that utilizes a variety of biopsychosocial supports [38].

## 3. Implications for Clinical Practice

Overall, this cumulative body of research on communicative participation in dysarthria provides several important insights that have implications for both clinical practice and clinical research. The first insight is that restricted communicative participation is not necessarily predicted by or dependent upon objective impairment or activity-based clinical measures, such as speech intensity or speech intelligibility [12,17,19,23,27]. What does appear to predict communicative participation is the individual’s perception of their speech production or perceived speech severity versus the results of objective measurement of the adequacy of speech [27]. To gain an accurate understanding of interferences to communicative participation, clinicians must ask their patients directly about their experiences rather than making inferences based only on objective clinical measures such as speech intelligibility, speech rate, or speech intensity, all of which may be inaccurate or misleading if these measures are used to predict interferences to communicative participation [40]. This finding has been demonstrated consistently across several studies examining predictors of communicative participation across a variety of diagnosis groups and dysarthria types [27,28,29,30,31]. The second insight is that communicative participation is influenced by multiple and complex variables, only some of which specifically reflect communication disorders and speech production [30,34]. Baylor and colleagues advocate that if the purpose of clinical management is to improve communicative participation, then clinicians may need to explore beyond the traditional boundaries of speech-language pathology by acknowledging and addressing other health issues, along with an individual’s unique personal, social, and physical environment [30]. Inclusion of a participation-based approach to management allows the clinician to see their patient through a broader lens, which can facilitate relevant and meaningful rehabilitation, and can enhance holistic care through the provision of referrals to other health care professionals if unmet needs are identified that fall outside of our scope of practice.

Finally, we would be remiss to ignore what individuals with dysarthria are telling us, especially in the context of what they view as constituting meaningful, clinically relevant, and ultimately successful clinical interventions. For example, Yorkston et al. interviewed individuals with PD who were asked to describe their experiences with speech treatment and share advice with clinicians on how to improve speech treatment [41]. The first piece of advice given by participants with PD was that clinicians should acknowledge that speaking is difficult, and the focus of interventions should include attention to the linguistic and cognitive demands of speaking. Next, drill work or speech exercises were not viewed positively, and these tasks and activities were abandoned unless they were tailored to the needs of the individual, tied to relevant and meaningful activities, and placed in the larger context of their disorder. Participants also stressed the importance of family support and education, but felt that clinical interventions did not adequately engage family members in the context of intervention. Finally, participants urged clinicians to place their speech production in the broader context of ageing with PD [41]. This study emphasizes that clinical interventions must be situated within a broader psychosocial context that addresses not only the motor speech aspects of dysarthria, but also other factors, such as physical effort, additional cognitive and emotional resources, and issues related specifically to the disease or disorder such as mobility and fatigue, all of which can impact communicative participation [41].

### 3.1. Development of Outcome Measures

Although there are numerous outcome measures that focus on quality of life and psychosocial functioning, only two outcome measures appear to specifically address the construct of communicative participation: the Communicative Participation Item Bank, 10-item short form (CPIB) [26], and the Communicative Effectiveness Survey (CES) [12]. It is essential to measure communicative participation specifically because it is a construct distinct from quality of life and other psychosocial measures, such as well-being [40]. Baylor cautions that measures of quality of life are not accurate indicators of communicative participation, therefore communicative participation cannot be inferred from psychosocial outcomes, including quality of life [40]. To address this need, Baylor et al. created an outcome measure specific to the construct of communicative participation. The CPIB short form is a 10-item, disorder-generic instrument developed for community-dwelling adults with communication disorders [26]. The items contained on the CPIB provide a patient-reported assessment of the extent to which their communication disorder interferes with communicative participation across a variety of speaking situations including, but not limited to, talking on the telephone to obtain information, ordering a meal at a restaurant, and communicating when needing to say something quickly. Items on the CPIB are rated on a four-point scale; a rating of 3 represents that the communication disorder does not at all interfere with participating in a particular speaking situation, whereas a rating of 0 represents that the communication disorder very much interferes with participating in a particular speaking situation. An advantage of the CPIB is that the summary score (ranging from 0–30) can be converted to item response theory theta values and/or standard *T* scores (*M* = 50, *SD* = 10). The CPIB has been validated for use with individuals with PD, ALS, MS, head and neck cancer [26], SD [42], and aphasia [43], and it is a valid and sensitive measure of pre-post treatment effects [44,45]. The CPIB can also be administered via Computer Adaptive Testing. Information about online administration can be found at CPIB Resource ePortfolio (https://sites.google.com/uw.edu/cpib/home accessed on 16 February 2022) [46].

The Communicative Effectiveness Survey, created by Donovan and colleagues, is an eight-item questionnaire focusing on communicative effectiveness, rated on a four-point Likert scale. A rating of 1 represents that communication is not effective, and a rating of 4 represents that communication is very effective across various speaking situations and contexts, such as having a conversation while traveling in a car, being part of a conversation in a noisy environment, and conversing at a distance, to name a few examples [12]. The CES permits individuals with dysarthria and their communication partners to rate perceived communicative effectiveness in various communicative situations/contexts to identify which are perceived as most difficult.

Both the CPIB and CES measure communicative participation and have the advantage of being relatively brief measures to administer during a research protocol or clinical visit. These patient-reported outcome measures provide quantitative clinical data that can aid the clinician and researcher in quantifying the severity of dysarthria from a broader perspective, and can provide valuable patient-reported information that can assist in guiding treatment planning, goal setting, and assessment of treatment outcomes.

Based on themes common to several studies exploring predictors of communicative participation, it appears as if speech usage and perceived speech severity are important predictors of communicative participation. Although not a specific participation-based outcome measure, as is the case for the CPIB or CES, the Levels of Speech Usage (LSU) is a self-report categorical rating scale that can be administered to describe and code the speech usage of people with a range of communication disorders in both clinical and research settings [47]. The LSU describes speech in terms of the amount, frequency, type, and importance of speaking situations that people might encounter without reference to specific occupations [47]. Individuals are asked to rate their speech usage in one of five categories: undemanding, intermittent, routine, extensive, and extraordinary. Clinicians can use the LSU scale as an initial starting point in which to frame their patients’ specific speech needs and priorities [47]. Gathering information on speech usage is important because it can provide additional context in which to plan participation-based interventions. Several studies have also revealed that perceived speech severity is an important predictor of communicative participation. The ‘speech’ subscale of the Amyotrophic Lateral Sclerosis-Functional Rating Scale (ALS-FRS) [48], was used by McAuliffe et al. to obtain self-rated perceived speech severity for participants with PD [27]. Using the speech subscale of the ALS-FRS, individuals are asked to choose one statement, from a list of five, that best describes their perceived speech intelligibility. Responses range from ‘normal’ through to ‘not understandable—I do not use speech for communication’ [48]. Information on perceived speech severity can also be gathered informally during a clinical interview, or patients could be asked to rate their perceived speech severity on a 100 mm visual analogue scale with the anchors ‘normal’ and ‘severely abnormal/impaired’. The patient’s perceived speech severity can be compared to objective clinical measures to determine if perceived speech severity aligns or does not align with objective ratings of speech severity, such as a speech intelligibility measure. Similar to the LSU scale, obtaining information on perceived speech severity can also be gathered and used as initial starting point for a discussion related to patients’ specific speech needs and priorities. Obtaining information on perceived speech severity is important because it can provide additional context in which to plan participation-based interventions.

### 3.2. Approaches to Intervention

One of the challenges of incorporating participation-based interventions in clinical practice is the relative absence of systematic protocols. As a result, many clinicians report that they do not have the requisite knowledge or skills to operationalize participation-based intervention into their daily clinical practices [49]. In response to this research-to-practice gap, Baylor and Darling-White proposed a comprehensive roadmap to provide concrete guidance to clinicians for conducting participation-based assessments, generation of goals, outcome measurement and documentation, and individualized and holistic interventions [49]. The main tenets of their philosophy center on person-centered care, shared patient-clinician decision making, and, ultimately, the provision of value-based clinical services that can be applied across many communication disorders and across the lifespan. Baylor and Darling-White stress that participation-focused intervention must begin with an understanding of the wants and needs of the individual with respect to their daily communication, and ends when the individual has gotten as close as possible to experiencing their desired communication wants and needs [49]. Using a modified organization of the WHO ICF conceptual framework, Baylor and Darling-White placed communicative participation as the central focus of treatment planning and implementation, with communication skills (ICF impairment and activity), the communication environment (ICF environmental factors), and personal perspectives (ICF personal factors) as factors contributing to and interacting with communicative-participation. Using this framework, a specific communicative participation goal is co-created with the patient (i.e., shared patient-clinician decision making), and is written so that the goal is observable and measurable within a discrete time period. Once the goal is defined and written, the next step is to address the specific communication skills, the communicative environment, and personal perspectives. An assessment of each of these three components is conducted, using either formal or informal measures, then specific goals are written to target the communication skill (e.g., based on targeting rate of speech, speech intelligibility, compensatory strategies), the communicative environment (e.g., reducing environmental barriers such as noise, goals to manage the physical or social environment), and personal perspectives (e.g., goals to address how patients feel about their participation, goals addressing the implementation of coping strategies). Finally, intervention will address systematically the targeted communication skills (e.g., slowing rate of speech to improve conversational speech intelligibility), how specific modifications to the environment can be achieved (e.g., self-advocacy, reduction of background noise), and how personal perspectives such as coping skills can be targeted by integrating counseling into our clinical interventions. Once the intervention has addressed these three components, then the final step is to measure these outcomes from the perspective of the progress or success of advancing the overall participation goal [49]. Some important caveats emerge from this research. The first is that communicative participation-focused intervention must involve assessing communicative participation as a distinct construct. Upholding a traditional impairment and activity-based intervention focus with the hope that this type of intervention will translate to improved communicative participation is misguided [49]. Traditional impairment-based approaches to intervention does not guarantee a translation to successful communicative participation. Relatedly, the second caveat is that, when providing interventions addressing personal perspectives, it should never be assumed that an individual’s emotional reaction as the result of their communication disorder is proportionate to the severity of their communication disorder or physical impairment [49]. Finally, the third caveat is that patient-reported outcome measures should be the primary indicator of assessing participation restrictions, and these patient-reported outcome measures should be used to document and measure intervention outcomes [49]. Goal attainment scaling (GAS) is an excellent option for goal setting and measuring meaningful levels of progress or success. GAS is a standardized, but person-centered, goal format in which levels of success or progress are rated by the patient on a five-point scale where -2 represents the worst possible outcome, 0 represents the most likely outcome, and +2 represents the best possible outcome [49,50]. For readers wishing to see how participation-focused intervention is operationalized within clinical practice, Baylor and Darling-White have provided concrete examples of participation-focused interventions using a shared patient-clinician decision-making approach for adults, adolescents, and children [49].

## 4. Conclusions

The cumulative body of knowledge informing our understanding of interferences to communicative participation in the dysarthrias underscore that people with dysarthria experience significant restrictions to communicative participation in their daily lives, that these restrictions cannot be inferred from objective speech measures, and that treatment goals and treatment outcomes focused specifically on improving communicative participation should be incorporated into clinical interventions. This body of knowledge also facilitates clinical research, particularly when exploring the outcome of interventions designed to address psychosocial aspects of communication disorders [41]. Although traditional speech interventions are indispensable in the clinical management of dysarthria, we argue that communicative participation should be a primary focus of treatment planning and intervention if it is our goal to provide patient-centered, holistic, and value-based clinical interventions which are responsive to the needs of individuals living with dysarthria. As speech-language pathologists, we are trained communication specialists, and in our unique role, we must advocate for the patients we serve through clinical interventions that embody a comprehensive and holistic approach to care. The inclusion of communicative participation outcomes in our clinical practice and clinical research can provide insight into the patient’s perceived level of function, the perceived severity of their communication disorder or disease, ensures the development of holistic, meaningful, and relevant clinical goals and outcomes, and provides a much-needed additional frame of reference when evaluating clinical outcomes for individuals with dysarthria.

## Data Availability

Not applicable.
